# Supplementary material for: Block Copolymer Adsorption on the Surface of Multi-Walled Carbon Nanotubes for Dispersion in N,N Dimethyl Formamide
Source: Nanomaterials (Basel). 2023 Feb 23;13(5):838. doi: 10.3390/nano13050838 (PMC10004759; doi:10.3390/nano13050838)
Supplement: Supplementary file 1 [file nanomaterials-13-00838-s001.zip › nanomaterials-2199462-supplementary.pdf]

Block Copolymer Adsorption on the Surface of Multi-Walled Carbon Nanotubes for Dispersion in *N,N* Dimethyl Formamide

Irena Levin [1], Aurel Radulescu [2], Lucy Liberman [1] and Yachin Cohen [1,*]

[1] Department of Chemical Engineering, Technion-Israel Institute of Technology, Haifa 3200003, Israel
[2] Forschungszentrum Jülich GmbH, Jülich Centre for Neutron Science (JCNS-4) at Heinz Maier-Leibnitz Zentrum (MLZ), D-85747 Garching, Germany
* Correspondence: yachinc@technion.ac.il

Supplementary Materials

1. Verification of constant CNT content in MWCNT/polymer dispersions

The sample sets prepared for contrast variation measurements of small-angle neutron scattering (SANS) with contrast variation need to be verified for having a constant CNT concentration, since the model-fitting of the SANS patterns require absolute intensity values. As described in the manuscript the samples at the different contrasts were prepared by suitable mixing of dispersions in fully protiated and fully deuterated DMF, which were centrifuged before mixing in order to remove extraneous carbon material and large aggromerates. As this reduces the CNT content UV-vis spectra were measured to verify constant relative composition in all dispersions. Figure S1 presents the absorbance data of MWCMTs/ $S_{12}4VP_{12}$ dispersions thus prepared, at different DMF/DMF-d7 solvent compositions for SANS measurements at different contrasts.

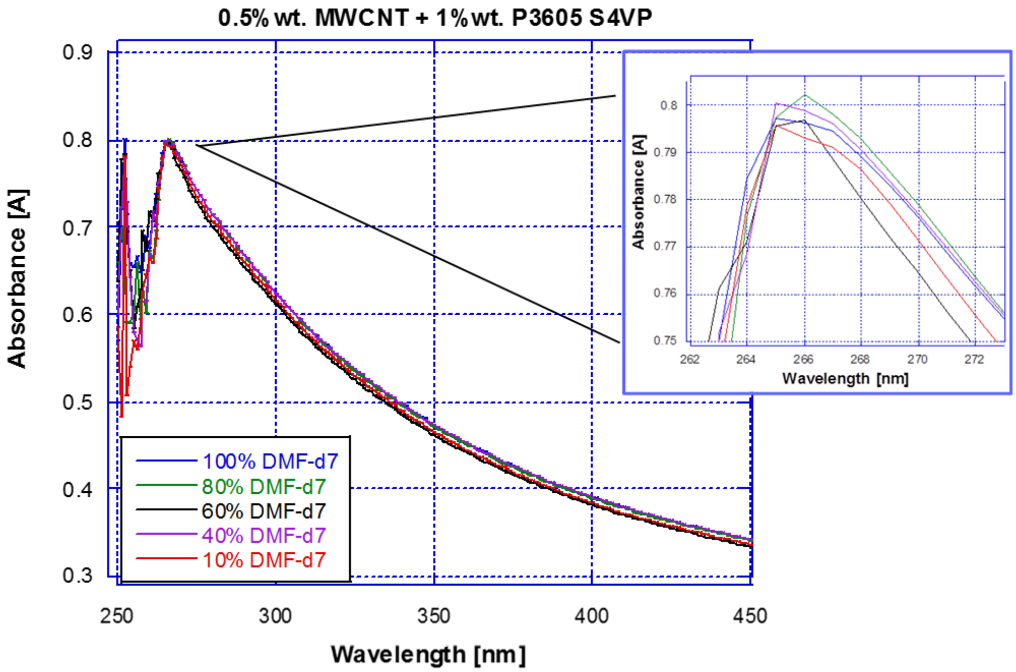

Figure S1*:* UV-vis absorbance of the 0.5%wt. MWCNT + 1%wt. $S_{12}4VP_{12}$ (P3605) dispersions at five contrasts. Insert shows magnification of the peak area.

2. SANS measurements of S_{12}-$4VP_{12}$ copolymer solutions.

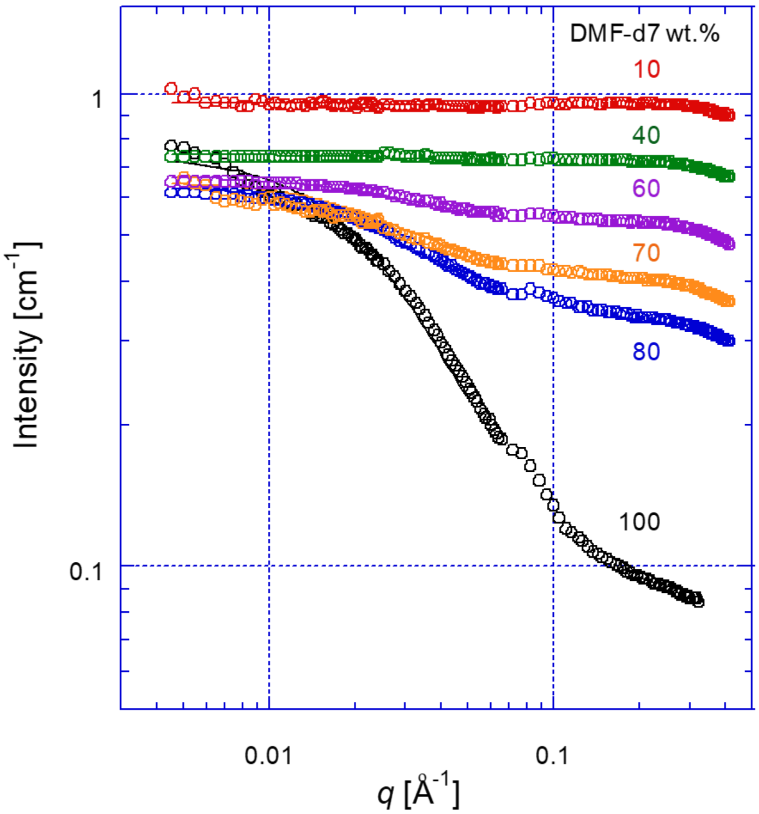

Figure S2*:* SANS measurements of S_{12}-$4VP_{12}$ copolymer solutions at different DMF-d7 solvent compositions.

3. SANS measurements of $dS_{11.5}$-$4VP_{11.3}$ copolymer solutions

SANS measurements of the copolymer of deuterated styrene block (11.5 kDA) and 4-vinyl pyridine block (11.3 kDa), at different contrasts, using solvent mixtures of deuterated DMF-d7 and DMF, listed in the caption to Figure S3.

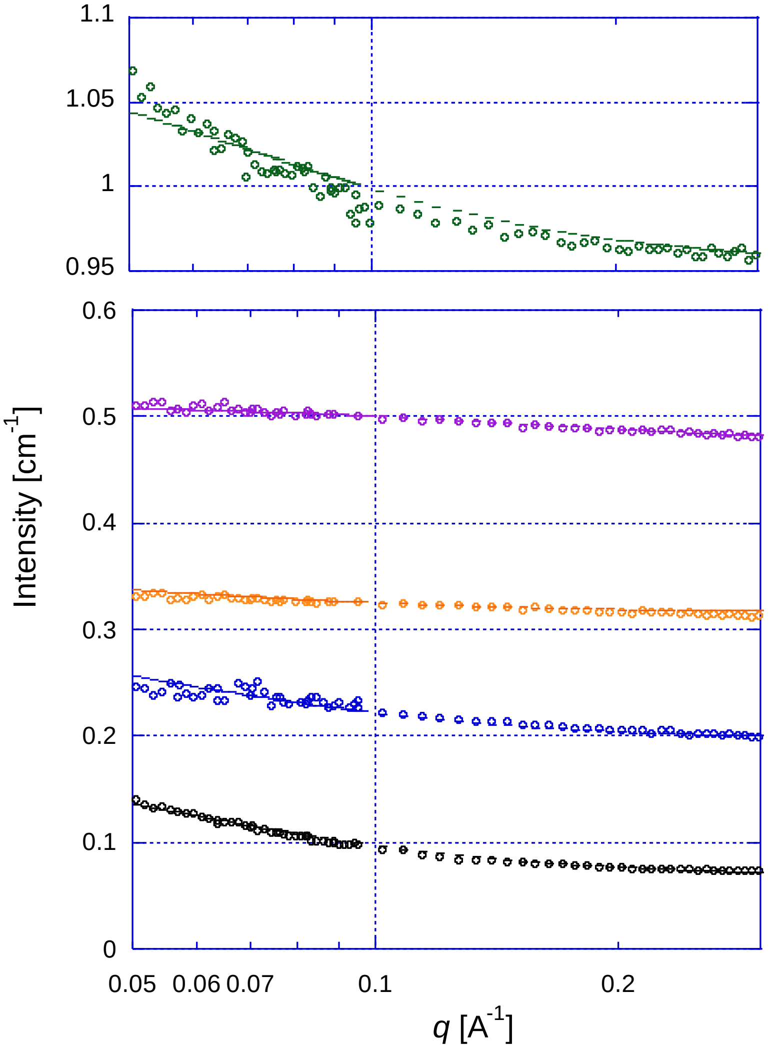

Figure S3: SANS measurements of dS11.5-4VP11.3 copolymer solutions at different DMF-d7 solvent compositions. Top to bottom: 10, 50, 70, 85, 100 wt.% DMF-d7. Circle: data; dashes: fit of Lorentzian function (eq. 1 in the manuscript)

3.1 Parameters used in analysis of the SANS measurements of $dS_{11.5}$-$4VP_{11.3}$ copolymer solutions.

Table S1. The parameters used to calculate the values of Y in the three equations (3) of the manuscript text, at three solvent contrasts (10, 85 and 100 wt.% DMF-d7).

Solvent composition (wt.% DMF-d7):	10	85	100
SLD (10^{-6} Å^{-2}) (1)	1.26	5.48	6.33
K_{PS} (10^{-6} Å^{-2}) (2)	5.03	0.81	-0.04
K_{P4VP} (10^{-6} Å^{-2}) (3)	0.61	-3.61	-4.46
K_C (10^{-6} Å^{-2}) (4)	2.84	-1.38	-2.23
Y (5)	0.893	-0.295	0.009
$\langle R_g^2 \rangle_{app}$ (Å^2) (6)	1253	873	1497

(1) SLD scattering length density
(2) $K_{PS} = SLD_{dPS} - SLD_{sol}$ (SLD difference between deuterated PS and solvent, at each contrast)
(3) $K_{P4VP} = SLD_{P4VP} - SLD_{sol}$ (as in (2) above, for P4VP)
(4) $K_C = K_{PS}W_{PS} + K_{P4VP}(1-W_{PS})$; (as in (2,3) above, for the entire copolymer)
W_{PS} = molecular weight faction of dPS: 11.5/(11.5+11.3)
(5) $Y= W_{PS}\, K_{PS}/ K_C$
(6) $\langle R_g^2 \rangle_{app}$ the apparent radius of gyration, as obtained from the fit of equation (2) in the manuscript, for each solvent compositions (contrasts).

The numerical values of equations (3) in the manuscript:

$\langle R_g^2 \rangle_{app} = \mathrm{Y}\langle R_g^2 \rangle_{PS} + (1-Y)\langle R_g^2 \rangle_{P4VP} + Y(1-Y)\,\langle L_o^2 \rangle$ (eq. 3 of manuscript)

are:

$$1497 = 0.009\langle R_g^2 \rangle_{PS} + 0.991\langle R_g^2 \rangle_{P4VP} + 0.00892\,\langle L_o^2 \rangle$$

$$873 = -0.295\langle R_g^2 \rangle_{PS} + 1.295\langle R_g^2 \rangle_{P4VP} - 0.382\,\langle L_o^2 \rangle$$

$$1253 = 0.893\langle R_g^2 \rangle_{PS} + 0.107\langle R_g^2 \rangle_{P4VP} + 0.0955\,\langle L_o^2 \rangle$$

The solutions of which are:

$\langle R_g^2 \rangle_{PS}^{0.5} \approx 32$ Å, $\langle R_g^2 \rangle_{P4VP}^{0.5} \approx 38.5$ Å and $\langle L_o^2 \rangle^{0.5} \approx 44$ Å .

4. Comparison of SANS and SAXS measurements of MWCNT/S_{12}-$4VP_{12}$ dispersions in 10 wt.% DMF-d7 solvent, and fit of the core-shell cylinder model.

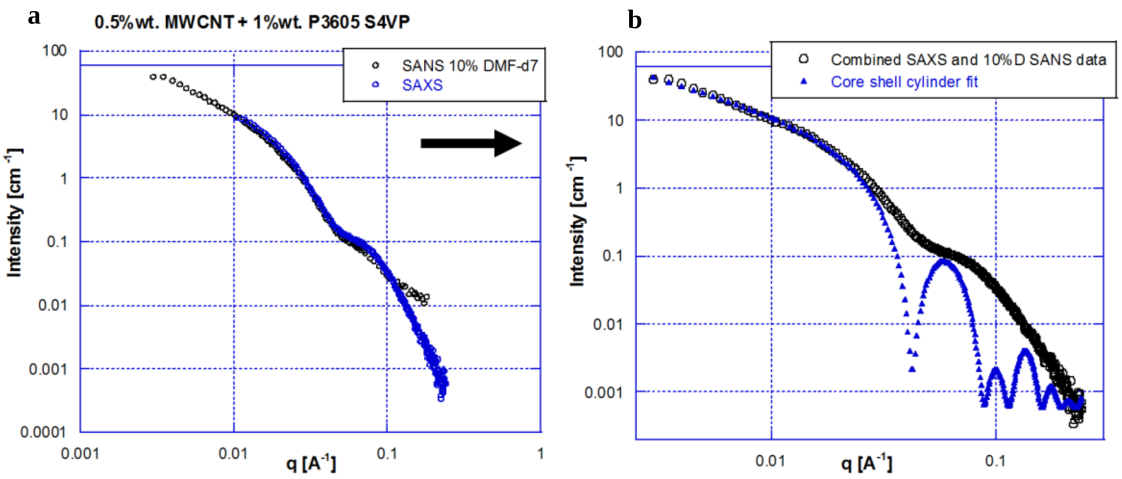

Figure S4: Scattering patterns of 0.5 wt.% MWCNT + 1 wt.% S_{12}-$4VP_{12}$ dispersions in 10 wt.% DMF-d7 solvent dispersion obtained by: (a) SANS and SAXS (SAXS data adjusted to the SANS intensity scale); (b) fit of the core-shell cylinder model to the SAXS data (one adjustable parameter: *n*.

5. Validation of the core shell cylinder model parameters for the 0.5 %wt. MWCNT + 1%wt. S_{12}-$4VP_{12}$ dispersions

The data at intermediate contrasts (40, 50, 70, 80 wt.% DMF-d7 solvents) were used to validate the model parameters obtained by fitting the measurements at 10 and 100% DMF-d7 solvent (Tables 4 and 5 in the manuscript). The requirement was that now the data should be consistently fitted without any adjustable parameters. Using the relevant SLDs at each contrast, the scattering intensity was calculated using the core shell cylinder model. The SLD of the MWCNT was calculated, as before, using the dimensions and solvent filling factor determined from the fitting of the 10% DMF-d7 data, as discussed in the manuscript text.

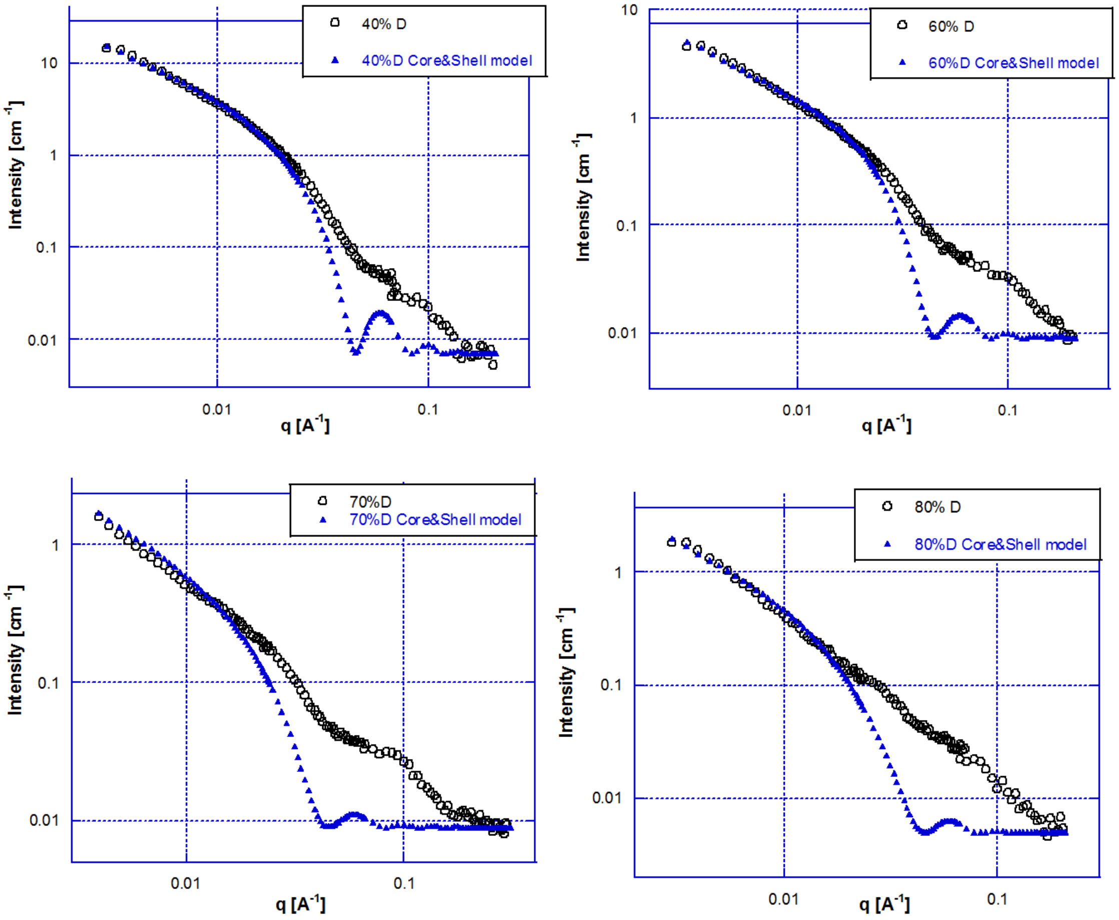

Figure S5: Validation of the core shell cylinder model parameters: SANS patterns of the 0.5 %wt. MWCNT + 1%wt. S_{12}-4VP_{12} dispersions in 40, 60, 70 and 80% DMF-d7 solvent, with the model calculations, without adjustable parameters.

Table S2. SLD values used in the core-shell cylinder model calculations at intermediate contrasts for parameter validation (Figure S5).

DMF-d7 wt.% in solvent mixture	40%	60%	70%	80%
Solvent SLD [10^{-6} Å^{-2}]	5.94	6.03	6.07	6.12
MWCNT core SLD [10^{-6} Å^{-2}]	2.94	4.05	4.61	5.17
Polymer shell SLD [10^{-6} Å^{-2}]	2.95	4.08	4.64	5.20

6. Validation of the core shell cylinder model parameters for the 0.5 %wt. MWCNT + 1%wt. dS$_{11.5}$-4VP$_{11.3}$ dispersions

The data at intermediate contrasts (50, 70, 85 wt.% DMF-d7 solvents) were used to validate the model parameters obtained by fitting the measurements at 10 and 100% DMF-d7 solvent (Table 7 in the manuscript), as previously done for the **MWCNT/**S$_{12}$-4VP$_{12}$ dispersions, without any adjustable parameters.

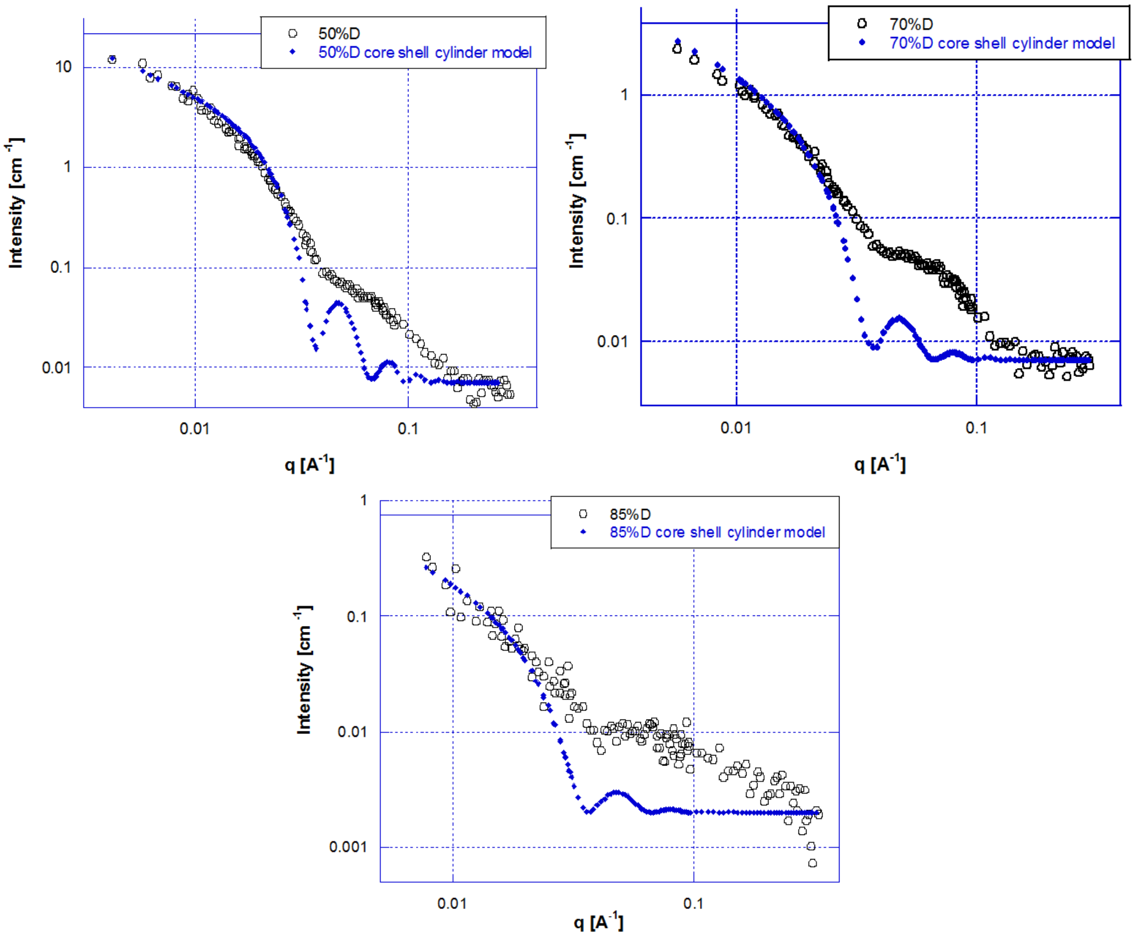

Figure S6: Validation of the core shell cylinder model parameters: SANS patterns of the 0.5 %wt. MWCNT + 1%wt. $dS_{11.5}$-$4VP_{11.3}$ dispersions in 50, 70 and 85% DMF-d7 solvent, with the model calculations, without adjustable parameters.

7. SANS patterns of solutions and dispersions in 10 wt.% DMF-d7 solvent using copolymers of different PS molecular weights.

SANS measurements of dispersions with the S_{124}-4VP_{12} and S_{48}-4VP_{11} were performed only with the 10 and 100 wt.% DMF-d7 solvent composition. Here the data at 10% DMF-d7 is presented. At the 10% DMF-d7 contrast, all the 3 block copolymers are nearly matched, therefore, their contribution to the scattering intensity is minimal as shown in Figure S7a. At this contrast, the dominant contribution to the scattering intensity from the polymer solutions is due to the incoherent scattering from hydrogen atoms. The scattering from the MWCNTs dominates the patterns from the hybrid dispersions, as shown in Figures S7b and c, before and after background subtraction, respectively, where the scattering patterns of all three hybrid types almost overlap in the entire q range.

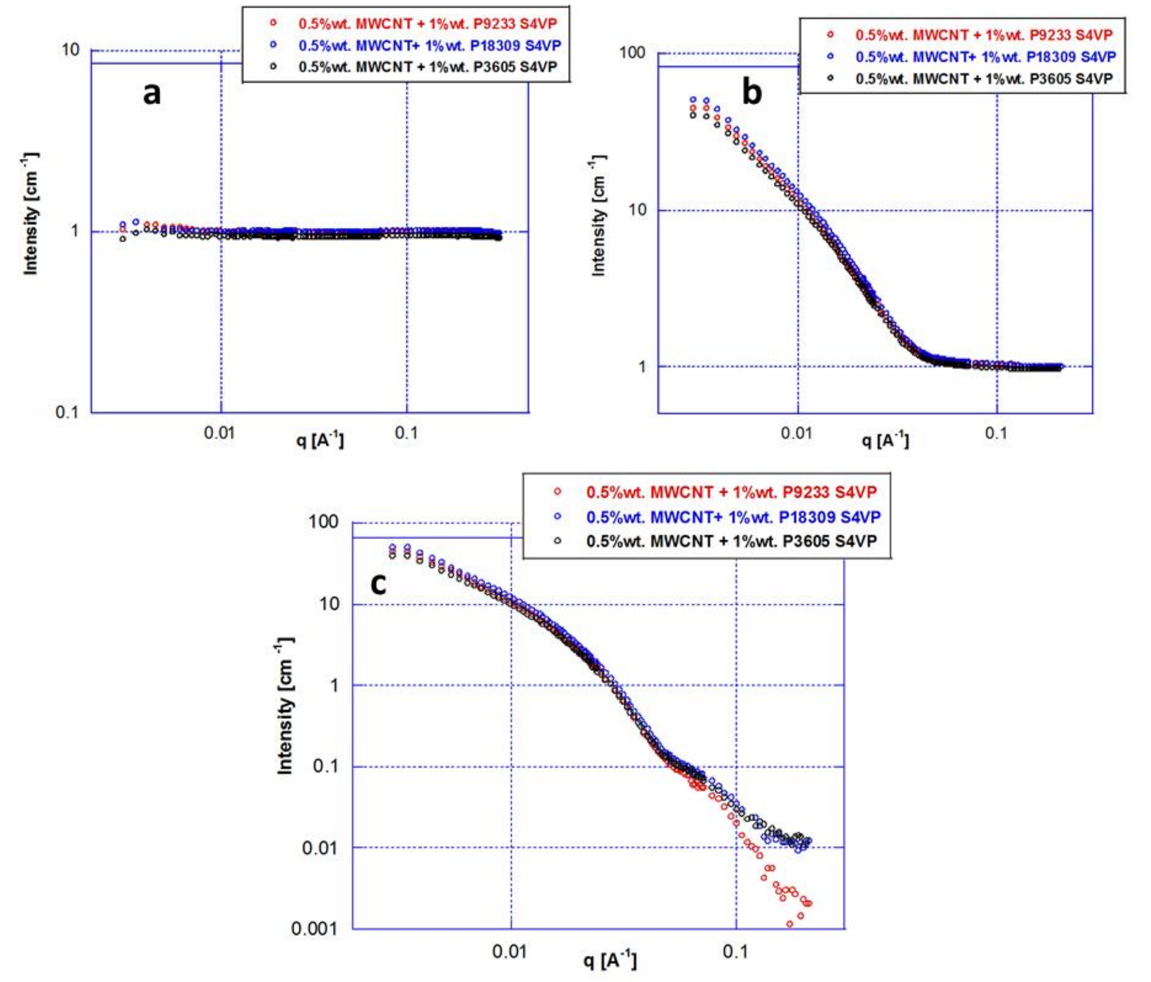

Figure S7: SANS patterns of solutions and dispersions in 10 wt.% DMF-d7 solvent using copolymers of different PS molecular weight: (a) solutions of S_{12}-4VP_{12} (black), S_{48}-4VP_{11} (blue) and S_{124}-4VP_{12} (red) copolymers; (b),(c) MWCNT/polymer dispersions before and after background subtraction, respectively.
